# The Innate Immune Response Characterizes Posterior Reversible Encephalopathy Syndrome

**DOI:** 10.1007/s10875-021-01033-3

**Published:** 2021-04-12

**Authors:** Christopher Nelke, Andreas Schulte-Mecklenbeck, Marc Pawlitzki, Leoni Rolfes, Saskia Räuber, Catharina C. Gross, Jens Minnerup, Sven G. Meuth, Heinz Wiendl, Tobias Ruck

**Affiliations:** 1 Department of Neurology with Institute of Translational Neurology, University of Münster, Albert-Schweitzer-Campus 1, D-48149 Münster, Germany; 2 Department of Neurology, Medical Faculty, Heinrich-Heine University Düsseldorf, Düsseldorf, Germany

**Keywords:** PRES, posterior reversible encephalopathy syndrome, flow cytometry, immune cells

## Abstract

While posterior reversible encephalopathy syndrome (PRES) is often characterized by an inflammatory cerebrospinal-fluid (CSF) profile, knowledge of immune cell patterns in PRES is lacking. Thus, we retrospectively characterized CSF and peripheral blood (PB) from 15 PRES patients, which we analyzed by multidimensional flow cytometry (FC). Results were compared to 72 controls, as well as to 9 patients with progressive multifocal leukoencephalopathy (PML, as a relevant differential diagnosis) and 15 multiple sclerosis patients (MS, as a classical neuroinflammatory disorder), respectively. Total protein level in CSF from PRES patients was elevated compared to that in controls, but not to MS and PML. In-depth FC analysis revealed no differences for adaptive immune cells (B cells, plasma cells, CD4^+^, and CD8^+^ T cells) in PB or CSF of PRES compared to controls. In contrast, we observed alterations of the adaptive immune response in CSF of PML and MS compared to PRES, indicating that the adaptive immune response is not a driver of disease in PRES. Indeed, PRES was characterized by an innate immune response with CD14^++^/CD16^+^ (intermediate) monocytes elevated in PB and CSF, while CD14^++^/CD16^−^ (classical) monocytes were decreased in PB from PRES patients as compared to controls. Levels of CD14^++^/CD16^+^ monocytes correlated with the duration of hospital stay as a surrogate marker for disease severity in PRES patients. Our findings argue for a role of innate rather than adaptive immunity in the pathophysiology of PRES. The observed shift in monocyte subsets might provide valuable diagnostic clues for the clinical management of these patients.

**Supplementary Information:**

The online version contains supplementary material available at 10.1007/s10875-021-01033-3.

## Introduction

Posterior reversible encephalopathy syndrome (PRES) is a heterogeneous clinico-radiological entity with characteristic clinical presentation including headache, visual disturbances, impaired consciousness, and seizures [1, 2]. The diagnosis is usually established by concurrent vasogenic edema within the occipital and parietal regions on magnetic resonance imaging (MRI) [3]. PRES remains a rare condition, and studies estimating the incidence are still lacking [2]. Common factors associated with PRES are hypertension, eclampsia, renal or liver failure, autoimmune disease, severe infection, drug abuse, or immunosuppressive medication, being present alone or in combination [1, 4]. The clinical heterogeneity in presentation and resulting insufficient diagnostic certainty render PRES a therapeutic challenge, particularly as differential diagnoses in patients under immunotherapy, such as progressive multifocal leukoencephalopathy (PML), can display overlapping clinical and/or radiographic features mimicking PRES [5, 6]. Differentiating PRES from PML might be very challenging with immunosuppression as a preceding factor and characteristic radiological features seen on presentation shared between both pathologies [6]. However, accurate diagnosis is crucial for initiation of appropriate treatment and, ultimately, preserving neurological function. While assessment of cerebrospinal fluid (CSF) might provide diagnostic clues, only a small number of studies investigated CSF from PRES patients reporting elevated CSF protein levels and concomitant normal cell numbers as a common finding in these patients [7, 8]. As CSF protein levels correlate with the extent of cerebral edema, protein levels are likely a manifestation of blood-CSF-barrier (BCSFB) impairment. Besides elevated protein levels, pleocytosis was frequently observed in CSF of PRES patients, indicating an inflammatory CSF profile [9]. However, in-depth analysis of immune cell patterns is lacking.

We here aim to characterize immune cell patterns in PB and CSF of PRES patients to further characterize the inflammatory CSF profile and enhance the diagnostic workup available for PRES. Our results suggest that multidimensional flow cytometry of CSF and blood could be a beneficial tool in the diagnostic workup of PRES, which might facilitate prompt diagnosis and initiation of appropriate therapy.

## Methods

### Study Design and Participants

Our study is a retrospective analysis of patients from a single center (University Hospital Muenster, Germany) with a median follow-up of 16.3 months (interquartile range (IQR) 12.9) after diagnosis. Patients were identified by searching the on-site database. We identified 44 patients diagnosed with PRES.

For this study, the following criteria for the diagnosis PRES were required:

- At least one characteristic clinical feature (i.e., headaches, altered consciousness, visual disturbances, seizures)
- Characteristic MRI findings (MRI with bilateral areas of white matter edema in the posterior cerebral hemispheres)
- At least one repeat MRI showing resolution of neuroimaging findings (reduction of edema, but not complete remission of neuroimaging findings was required [7])
- Absence of a viable differential diagnosis (i.e., diffusion-weighted imaging was performed for all patients).

**Fig. 1:**
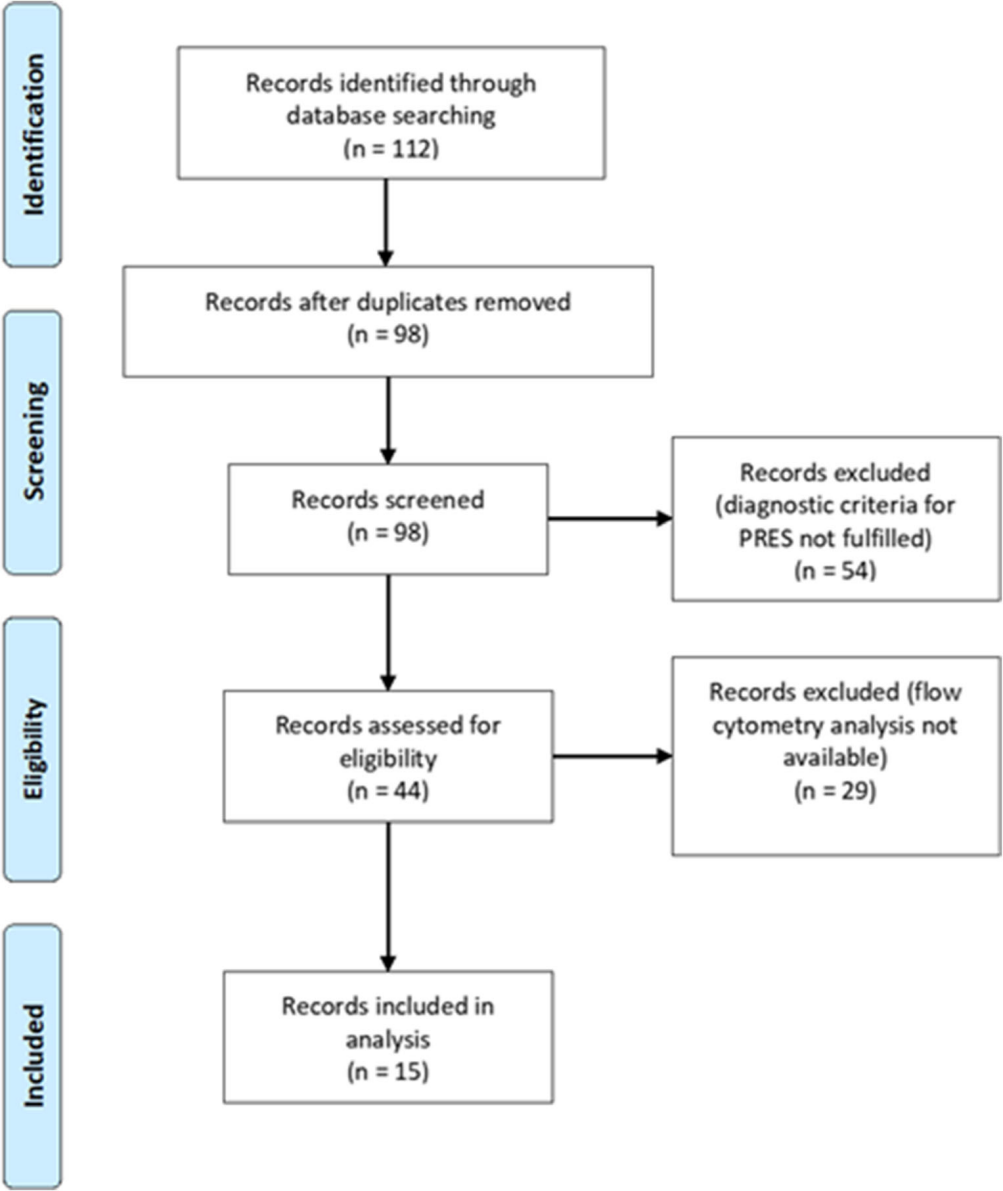
PRISMA flow chart detailing screening and inclusion of patient records for this study

Out of those 44 patients diagnosed with PRES, FC data from CSF and peripheral blood (PB) were available in 15 cases (Fig. 1). Clinical and demographic data on patients included in the final analysis can be found in the Supplementary Table 1 and Supplementary Table 2. All patients were treated at the University Hospital Muenster between 2014 and 2020. CSF was additionally acquired from a hospital-based cohort of 72 controls. In these individuals, the presence of a neurological disorder had been suspected but was not confirmed. In addition to the clinical classification, patients serving as control were required to have an MRI workup without pathological findings. CSF for controls was required to fulfill the following laboratory criteria defining a non-inflammatory CSF (< 5 cells/μl, < 500 mg protein/ml, < 2 mmol/l lactate, no disruption of the blood/CSF barrier, no oligoclonal bands (OCB) in the CSF, and no intrathecal immunoglobulin (Ig) G, IgA, or IgM synthesis) [10, 11].

**Table 1:** Clinical and demographic characteristics of patients

Characteristic	PRES (*n* = 44)	PRES patients with FC available (*n* = 15)	Controls (*n* = 72)
Sex (% female)	75%	60%	65.2%
Age at first manifestation, years (IQR)	64 (29.8)	61 (21)	34.3 (25.9)
Systolic blood pressure at admission, mmHg (IQR)	145 (40)	140 (40)	125 (30)
Diastolic blood pressure at admission, mmHg (IQR)	90 (17.5)	90 (20)	85 (25)
Length of hospital stay in days, median (IQR)	10 (8)	10 (9)	5 (6)
Trigger			
Hypertension	20 (45.4%)	6 (40.0%)	
Renal or liver failure	11 (25.0%)	4 (26.7%)	
Infection	11 (25.0%)	4 (26.7%)	
Adverse effect of immunotherapy	3 (6.8%)	1 (6.7%)	
Unknown	10 (22.7%)	3 (20.0%)	
Presenting neurological symptoms			
Headache	27 (61.3%)	5 (33.3%)	
Visual disturbances	25 (56.8%)	5 (33.8%)	
Altered consciousness	9 (20.4%)	4 (26.6%)	
Seizures	14 (31.8%)	3 (20.0%)	
Paresis	16 (36.3%)	4 (26.6%)	

Differences between groups were analyzed using Student’s *t* test or Mann-Whitney test. Unless otherwise reported, values are median ± IQR (range), median ± IQR, or *n* (%)

Abbreviations: *IQR*, interquartile range

**Table 2:** Routine CSF parameters

Characteristic	PRES (*n* = 15)	Controls (*n* = 15)	False discovery rate adjusted *p*-values
Total cells/μl	1 (0)	0 (0)	0.81
Lymphocytes/μl	1 (0)	0 (0)	0.95
Granulocytes/μl	0 (0)	0 (0)	0.87
Total protein in CSF (mg/l)	419 (326.5)	364 (290.0)	**<0.05**
Albumin in CSF (mg/l)	215 (166.5)	195 (183.0)	**<0.05**
Albumin in serum (g/l)	37.9 (32.5)	41.9 (38.7)	0.29
Albumin ratio	5.8 (5)	4.9 (3.6)	0.73
IgG in CSF (mg/l)	20.6 (12.5)	20.8 (18.8)	0.22
IgG in serum (mg/l)	7.7 (6.7)	9.1 (8.5)	0.88
IgA in CSF (mg/l)	2.5 (0.9)	2.2 (2.0)	0.81
IgA in serum (mg/l)	1.7 (1.4)	2.2 (1.6)	0.98
IgM in CSF (mg/l)	0.2 (0.1)	0.2 (0.1)	0.96
IgM in serum (mg/l)	0.7 (0.5)	1.8 (0.7)	0.98
Lactate (mmol/l)	1.9 (0.5)	1.1 (0.7)	0.96
Glucose ratio	0.63 (0.1)	0.63 (0.1)	0.99
BCSFB breakdown, *n* (%)	5 (33%)	0 (%)	0.98
Presence of oligoclonal bands in CSF, *n* (%)	2 (13%)	0 (0%)	0.98

Significance between groups was analyzed using unpaired Student’s *t* test or Mann-Whitney test as appropriate. To account for multiple comparisons, *p*-values were corrected by the false discovery rate. Significant findings are in bold. Unless otherwise reported, values are mean (±SD)

Abbreviations: *BCSFB*, blood-CSF-barrier; *CSF*, cerebrospinal fluid; *Ig*, immunoglobulin

### Data Collection

Demographic, clinical and follow-up data were obtained from electronic institutional records and included age, sex, length of hospital stay, vital parameters (blood pressure), precipitating factors (e.g., infections, comorbidities, medication such as immunosuppressants), and standard laboratory data.

### CSF Analysis

CSF and PB were acquired by lumbar puncture and blood collection during clinical workup at the discretion of the treating physician, respectively. Only samples from PRES, PML, or multiple sclerosis (MS) patients were analyzed that were obtained prior to any treatment. For MS, only treatment-naïve patients with their respective first manifestation were included. To assure optimal sample quality, all samples were analyzed within 1 h of sampling. A Fuchs-Rosenthal chamber was used to assess the CSF cell count. Protein concentrations and immunoglobulin levels (IgG, IgA, and IgM) were measured by nephelometry. Protein and Ig concentrations were compared and a Reiber scheme was created to evaluate the integrity of blood-CSF-barrier (BCSFB). Isoelectric focusing and silver nitrate staining were used to detect oligoclonal bands. Blood-stained CSF was excluded from the analysis. For FC analysis, acquired CSF samples were centrifuged for 15 min at 300×*g*, and the supernatant was discarded. CSF cells were treated with VersaLyse (Beckman Coulter) in parallel to 100 μl EDTA blood for 10 min. After washing, staining was performed according to a previously established protocol [11, 12] using CD3 (UCHT1); CD4 (13B8.2); CD8 (B9.11); CD14 (RMO52); CD16 (3G8); CD19 (J3–119); CD45 (J.33); CD56 (C218); CD138 (B-A38); and HLA-DR (Immu-357) antibodies (all Beckman Coulter). Absolute cell counts were calculated according to the number of Flow Count Fluorospheres used as internal standard. For gating strategy, see Supplementary Fig. 1. A Navios flow cytometer (Beckmann Coulter) and the software Kaluza (version 2.1) were used for data analysis.

### Standard Protocol Approvals, Registrations, and Patient Consents

The collection and analysis of biomaterial was approved by the local ethics committee and institutional review board of the University of Muenster (2019-712-f-S). Data were anonymized and collected retrospectively.

### Statistical Analysis

Statistical analysis was performed using GraphPad Prism 8.4 (GraphPad Software, Inc., San Diego, CA) and SPSS 25.0 (IBM, Armonk, New York). Data were presented as median (IQR = interquartile range), mean (standard deviation = SD) or *n* (%). The D’Agostino–Pearson omnibus normality test was performed to test for Gaussian distribution. Differences between groups were analyzed using unpaired Student’s *t* test or Mann-Whitney *U* test as appropriate or Kruskal-Wallis test for multiple groups. Volcano plots were constructed by plotting log2 values of the relative difference between the medians (continuous) or means (categorical parameters) against the *p*-values, calculated using the Mann-Whitney *t*-test. To account for multiple comparisons, statistical significance was corrected by the false discovery rate (FDR) approach using a threshold of *Q* = 5%. For prediction of PRES, we included status of diagnosis as dependent variable in a multiple logistic regression model. For prediction of the length of hospital stay, we used a model of multiple linear regression with the former as dependent variable. Goodness-of-fit was assessed by Tjur’s *R* squared, significance by log-likelihood test. Significance was assumed if *p* < 0.05. Anonymized data will be shared by request from any qualified investigator.

## Results

### Clinical and Demographic Features

**Fig. 2:**
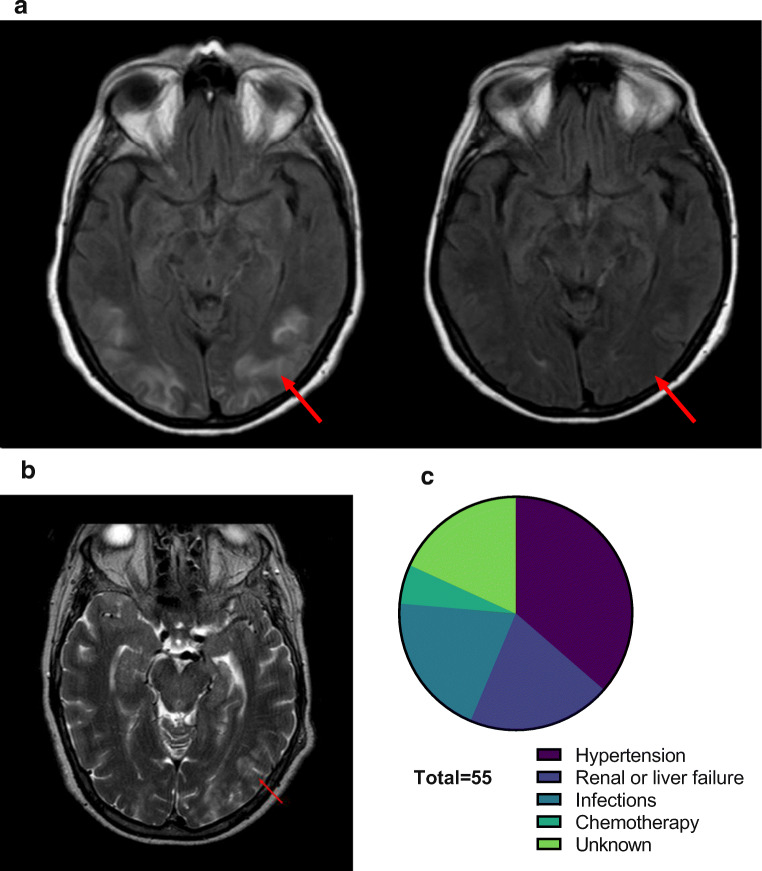
Radio-clinical findings of PRES patients. **a** Magnetic resonance imaging on T2 fluid-attenuated-inversion-recovery sequence displaying symmetric, bilateral edema with parieto-occipital dominance on the left hemisphere, and resolution of radiological findings after 2 weeks (arrow) in the same patient. **b** Magnetic resonance imaging on T2 fluid-attenuated-inversion-recovery sequence displaying unilateral edema with parieto-occipital dominance on the right hemisphere in a patient suffering progressive multifocal leukoencephalopathy as confirmed by biopsy. **c** Precipitating factors for the recorded cases of PRES

We included 44 patients with a mean follow-up of 16.3 months (SD ±12.9). Diagnosis was established according to clinical and radiological findings as described in the “Methods” section (Fig. 2a shows a characteristic case). The median age at manifestation of PRES was 64 years (IQR 29.8) with most patients being female (*n* = 33, 75%) (Table 1). On average, patients required a median hospital stay of 10 days (IQR 8). No deaths were recorded. Severe hypertension was the most common trigger for PRES in this study (*n* = 20, 45.4%) followed by renal or liver failure (*n* = 11, 25.0%) (Fig. 2c). Three cases were attributed to immunosuppressant medication or chemotherapy (6.8%). The remaining cases were associated with severe infections (*n* = 11 (25%)) or remained unknown (*n* = 10 (22.7%)). Systolic blood pressure at admission was elevated with 145 mmHg (IQR 40). Headache was the most frequent symptom on presentation (61.3% of cases) often with occipital accentuation, followed by visual disturbances (presentation included both hemianopsia and total vision loss, 56.8% of cases), paresis (36.3%), seizures (31.8% of cases), and altered consciousness (20.4% of cases). No in-hospital deaths were recorded. The median time between admission and sample collection was 1 day (IQR 0–2 days). The median age of patients serving as control was 34.3 years (IQR 25.9). There was a female predominance with 47 patients being women (65.2%).

### Total Protein Levels Are Elevated in CSF from PRES Patients

**Fig. 3:**
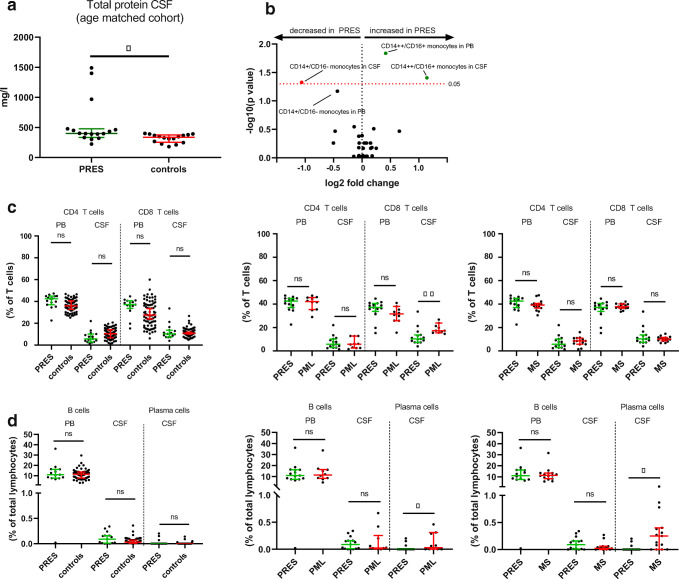
The adaptive immune response is unchanged in PRES. **a** Total protein levels in CSF from PRES and age-matched controls determined by Mann-Whitney test. **b** Volcano plot comparing PRES and controls constructed by plotting log2 values of the relative difference between the medians (continuous) against the *p*-values, calculated using the Mann-Whitney test. The dashed red line indicates *p* = 0.05 with points above the line are *p* < 0.05 and points below the line are *p* > 0.05. To account for multiple comparisons, statistical significance was corrected by the false discovery rate (FDR) approach. A threshold of *Q* = 5% was used for FDR. **c** Relative cell number for CD4^+^ and CD8^+^ T cells determined by Mann-Whitney test for control cohort (left), PML (middle), and MS (right). **d** Relative cell number for B cells and plasma cells determined by Mann-Whitney test for control cohort (left), PML (middle), and MS (right). For each comparison, PB is left, and CSF is right. Error bars display median (IQR). Abbreviations: CSF, cerebrospinal fluid; MS, multiple sclerosis; PB, peripheral blood; PML, progressive multifocal leukoencephalopathy; PRES, posterior reversible encephalopathy syndrome. ***p < 0.001, **p < 0.01, *p < 0.05

First, we analyzed routine CSF parameters. We compared total cell numbers as well as lymphocytes, granulocytes, and erythrocytes; total protein in CSF; albumin in CSF and serum; IgG level, IgA level, and IgM level in CSF and serum; lactate; and the frequency of BCSFB breakdown between PRES patients and controls (Table 2). Differences between groups were analyzed using Student’s *t* test or Mann-Whitney test. As expected based on previous studies [7, 9], total protein (median in mg/l (IQR); PRES 419 (362.5), control 360 (312.7), *p* < 0.001) and albumin (median in mg/l (IQR); PRES 215 (166.5), control 187 (153.7), *p* < 0.001) were elevated in the CSF of PRES patients (Fig. 3a). To account for demographic differences between groups, controls were matched by age and sex and the analysis was repeated accordingly. All results remained statistically significant (total protein in CSF, median (IQR); PRES 434 (394), control 368 (313.5), *p* < 0.05). Thus, we found elevated protein levels in CSF, whereas other routine parameters of CSF analysis were not significantly different.

We also compared total protein in CSF from PRES to PML (as highly relevant differential diagnosis) and MS patients (as a classical neuroinflammatory disorder), observing no difference between PRES and PML or MS (median in mg/l (IQR); PRES 419 (362.5), PML 599 (379), *p* = 0.09, MS 472 (392), *p* = 0.78).

### FC Analysis Reveals Characteristic Immune Cell Patterns in PRES Patients

**Fig. 4:**
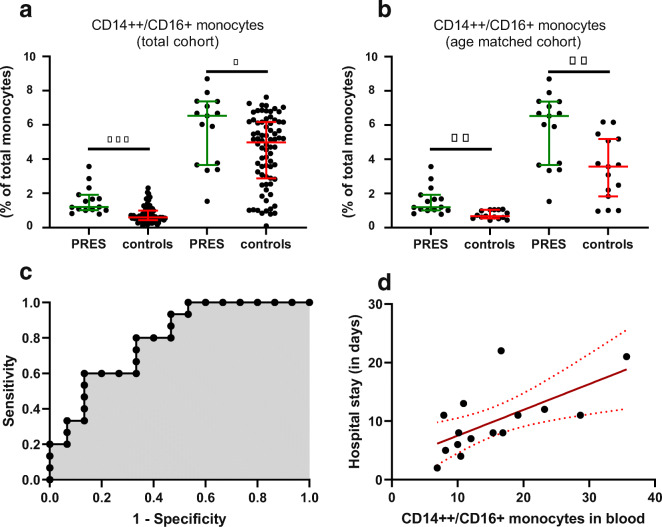
CD14^++^/CD16^+^ monocytes in PRES. **a** Relative cell number for CD14^++^/CD16^+^ monocytes determined by Mann-Whitney test for total controls. **b** Relative cell number determined for CD14^++^/CD16^+^ monocytes by Mann-Whitney test for age-matched controls. Left for PB, right for CSF. **c** Receiver operating characteristic (ROC) curve displaying logistic regression for relative CD14^++^/CD16^+^ monocyte numbers in peripheral blood as independent and diagnosis of PRES as dependent variable including PRES patients as well as age-matched controls. Significance was assessed by Log-likelihood test (OR 1.29 CI 1.08–1.70, *p* < 0.05, *R* = 0.22). **d** Linear regression displaying correlation of length of hospital stay (days) and CD14^++^/CD16^+^ cell number in PB as influencing variable. Abbreviations: PB, peripheral blood; PRES, posterior error lines display 95% confidence interval with *p* < 0.01 and *r*^2^ = 0.55

Next, as mild pleocytosis has been observed in PRES indicating an inflammatory CSF profile [8], we aimed to investigate the immune cell profiles of those patients (Fig. 3b). We assessed immune cell populations in CSF and PB of PRES patients by multidimensional FC analysis and compared our findings to controls. Furthermore, we chose PML as diseased control representing a neuroinflammatory disorder and relevant differential diagnosis, which is predominantly mediated by adaptive immunity. Thus, we included 9 patients presenting with PML verified by biopsy and 15 sex-matched, treatment-naïve multiple sclerosis (MS) patients representing a chronic neuroinflammatory process as well as to account for immune-modulating treatment as superimposed factor for PML. Clinical characteristics of MS and PML patients are displayed in Supplementary Table 2. First, we analyzed changes to cells belonging to the adaptive immune system. The population of T cells, including CD4^+^ T cells and CD8^+^ T cells (Fig. 3c), displayed no differences comparing PRES to controls or MS. In contrast, CD8^+^ T cells were elevated in CSF from PML patients. To investigate T cell activation, we analyzed HLA-DR^+^ CD4^+^ T and HLA-DR^+^ CD8^+^ T cells observing no changes to these cells comparing PRES to controls. Besides T cells, we characterized B cell numbers in PB and CSF of PRES. Compared to controls, PML, or MS, B cells remained unchanged. Furthermore, plasma cells were analyzed and remained unchanged between PRES and controls; however, elevated plasma cell numbers in CSF were observed in PML and MS (Fig. 3d). We also analyzed absolute cell numbers comparing PRES with controls, PML, and MS. Confirming our previous results, we observed no differences between PRES and controls for the adaptive immune response (Supplementary Fig. 2). In contrast, CD8^+^ T cells and plasma cells were elevated in CSF from PML and MS compared to PRES. Taken together, these findings indicate that the adaptive immune system is not a central driver of inflammatory changes in PRES pathophysiology. For innate immunity, characterization of total monocyte and granulocyte numbers displayed no alterations in PRES. As monocyte subsets are implicated in the propagation of vascular disease [13], we aimed to also characterize these populations in PRES. Indeed, analysis of monocyte subsets in CSF and PB of PRES patients revealed a population of intermediate monocytes expressing high levels of CD14 (CD14^++^) and low levels of CD16 (CD16^+^) to be more prevalent in the CSF and PB of PRES patients as compared to controls. In contrast, classical CD14^++^/CD16^−^ monocyte numbers were decreased in CSF (Fig. 3b). Age-dependent changes to monocyte numbers and distribution of subsets have been previously described in healthy controls [14]. To overcome the caveat of age as confounder, patients serving as controls were matched by age and sex to the PRES cohort and confirmed our previous results when accounting for age (Fig. 4b). We also investigated absolute cell numbers of CD14^++^/CD16^+^ monocytes in PB and CSF of PRES patients and aged-matched controls, which provided similar differences as seen for relative cell numbers (CSF: cells per milliliter, mean (SD); PRES 159.1 (164.7), control 57.7 (79.2), *p* = 0.02; blood: 1000 cells per milliliter, mean (SD); PRES 70.3 (58.8), control 24.8 (9.9), *p* < 0.01). Taken together, our findings delineate the presence of intermediate monocytes in CSF and serum of PRES patients and hint towards a shift in monocyte subpopulations in association with the disease.

### CD14^++^/CD16^+^ Monocytes Are Associated with Clinical Progression of PRES

Next, we aimed to deduce clinical value from the identification of intermediate monocytes in association with PRES. Given the diagnostic difficulty contiguously imposed by PRES, we investigated the ability of CD14^++^/CD16^+^ cells in PB to predict PRES by entering the relative CD14^++^/CD16^+^ cell number together with age and sex as clinical parameters in a model of multiple logistic regression. We additionally included CSF protein level to assess the additional value of CD14^++^/CD16^+^ cells compared to known markers. We chose the relative cell number, as normalization to beads is not required and clinical application might therefore be more robust. For analysis by logistic regression, we compared two models, one including the entire cohort of 72 patients serving as control and one including age-matched control patients, as we anticipated the difference in age between PRES patients and control as a confounder for regression analysis. Indeed, assessment by Tjur’s *R* squared revealed a superior *R* value for the age-matched cohort (age-matched *R* = 0.34, full cohort *R* = 0.22). In this model, relative CD14^++^/CD16^+^ cell number predicted the diagnosis PRES with significance assessed by the Log-likelihood test (OR 1.29 CI 1.08–1.70, *p* = 0.02) (Fig. 4c).

Next, we aimed to investigate whether CD14^++^/CD16^+^ cell numbers correlate with disease severity. As there are no established clinical measurements for assessing disease severity in PRES, we used the length of hospital stay as surrogate marker (median = 10 days (IQR 8)), particularly as this parameter has been previously used in association with intermediate monocytes [15]. As hospital stay is likely influenced by clinical parameters such as the number of comorbidities, we used a multiple regression model (Fig. 4d**)**. To exclude age and sex as confounder of clinical outcome, we employed a model of multiple linear regression including age, sex, the number of comorbidities, and the relative CD14^++^/CD16^+^ cell count in PB with the length of hospitalization as dependent variable. Here, only CD14^++^/CD16^+^ cells retained significance and were associated with the duration of hospital stay (*p* = 0.008 and *r*^2^ = 0.55, Fig. 4d). To also test the predictive value of total protein in CSF, we also included this parameter in the analysis and found no significant association between the length of hospitalization and total protein levels. Thus, CD14^++^/CD16^+^ monocytes are associated with the clinical course of PRES while also providing diagnostic clues.

Of note, two patients suffered residual neurological deficits. However, given the low number of patients with deficits, we did not consider residual symptoms a suitable read-out parameter for disease severity.

Lastly, to address the potential influence of immunological trigger underlying the development of PRES, we compared immune cell patterns in CSF and PB from cases likely due to immunological triggers (infections or immunosuppression) and cases triggered by other causes (Supplementary Fig. 3). Here, we observed no meaningful differences between the two groups. Statistical power might be impeded due to the lower number of patients in the group of immunological triggers.

### Monocytes Distinguish PRES from Other Neuroinflammatory Disorders

**Fig. 5:**
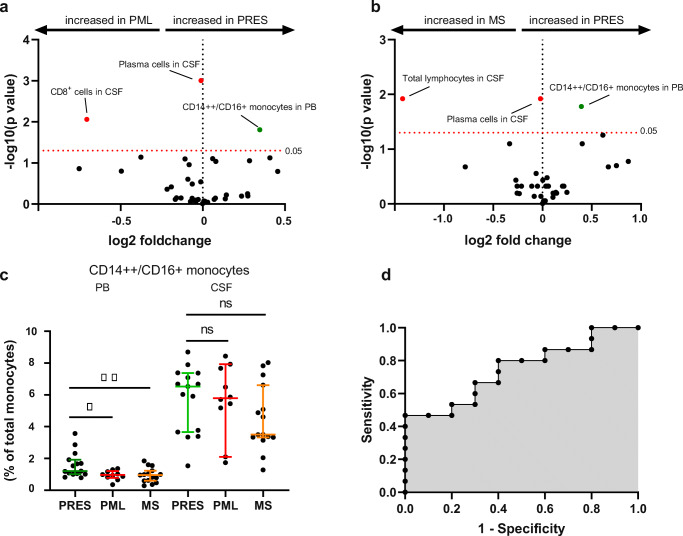
CD14^++^/CD16^+^ monocytes in PRES and PML. **a** Volcano plot comparing PRES and PML constructed by plotting log2 values of the relative difference between the medians (continuous) against the *p*-values, calculated using the Mann-Whitney test. The dashed red line indicates *p* = 0.05 with points above the line are *p* < 0.05 and points below the line are *p* > 0.05. **b** Volcano plot comparing PRES and MS constructed by plotting log2 values of the relative difference between the medians (continuous) against the *p*-values, calculated using the Mann-Whitney test. The dashed red line shows where *p* = 0.05 with points above the line having *p* < 0.05 and points below the line having *p* > 0.05. To account for multiple comparisons, statistical significance was corrected by the false discovery rate (FDR) approach. A threshold of *Q* = 5% was used for FDR. **c** Relative cell distribution determined for CD14^++^/CD16^+^ monocytes by Kruskal-Wallis test for age-matched cohort. Left for peripheral blood, right for CSF. **d** Receiver operating characteristic (ROC) curve displaying logistic regression for relative CD14^++^/CD16^+^ monocyte numbers in peripheral blood as independent and diagnosis of PRES as dependent variable for PRES and PML patients. Significance was assessed by Log-likelihood test (OR 1.3 CI 1.1–7.0, *p* < 0.01, *R* = 0.22). Abbreviations: CSF, cerebrospinal fluid; MS, multiple sclerosis; PRES, posterior reversible encephalopathy syndrome; PML, progressive multifocal leukoencephalopathy

Diagnosis of PRES constitutes a clinical challenge, as radiographic findings in PRES are often indistinguishable from other neuroinflammatory disorders such as progressive multifocal leukoencephalopathy (PML) (Fig. 2b shows an exemplary MRI) [6]. This conundrum is further aggravated by overlapping patient characteristics with immunosuppression described as preceding factor in both disorders [6]. Interestingly, we observed both plasma cells and CD8^+^ T cells to be elevated in CSF of PML compared to PRES (Fig. 5a). However, other cell types of the adaptive immune response comparing PRES with PML remained unchanged. For MS, we observed total lymphocytes and plasma cells to be increased in CSF from MS patients (Fig. 5b), in line with mild pleocytosis as characteristic finding in MS. Of note, changes to the innate immune response remain apparent in PRES when comparing to other inflammatory conditions. CD14^++^/CD16^+^ monocyte levels were elevated in PB compared to PML or MS (Fig. 5a and b). Next, we investigated whether CD14^++^/CD16^+^ monocyte levels in blood provides diagnostic clues for distinguishing PRES from other neuroinflammatory disorders. To this end, we entered the relative number of CD14^++^/CD16^+^ monocytes in blood from PRES and PML patients in a model of logistic regression. Here, CD14^++^/CD16^+^ monocytes predicted diagnosis of PRES (OR 1.3 CI 1.1–7.0, *p* < 0.01, *R* = 0.22) indicating that monocytes might hold value for improving the diagnostic approach to PRES.

## Discussion

The underlying pathophysiological mechanisms of PRES remain insufficiently understood, and diagnostic certainty is limited by the heterogenous presentation and dependence on coinciding clinico-radiological findings. Succinctly, our manuscript provides corroboration of previous studies [7, 8] reporting elevated protein levels in the CSF of PRES patients and evidence for the association of PRES with the monocyte composition shifting towards the intermediate subset (CD14^++/^CD16^+^). Additionally, in-depth FC analysis reveals no changes to cells of the adaptive immune system, such as T cells, B cells, or plasma cells in PRES. Together, as the first in-depth immune cell pattern analysis of PRES, our data argues for a role of innate rather than adaptive immunity in the pathophysiology of PRES.

Reflecting previous reports, we observed female predominance and advanced age for PRES patients [8, 9]. Moreover, our cohort corroborates hypertension, infections, and immunosuppressant medication as common trigger factors [1, 2, 9]. Indeed, hypertension was frequent with 45% of patients displaying elevated systolic blood pressure underlining the importance of this factor in the development of PRES. In contrast, seizures as presenting symptom were previously reported to be as frequent as 60% [9], while we observed seizures only in 30% of PRES patients, pointing towards clinical differences between PRES cohorts. Besides clinical findings, we also observed increased total protein levels in CSF of PRES patients. However, elevated protein levels in CSF are not disease-specific but were observed in a number of conditions affecting the central nervous system such as severe infections, autoimmune polyneuropathies, i.e., Guillain-Barre-syndrome, or lumbar stenosis [7, 16]. Moreover, we observed no difference for total protein levels when comparing PRES to potential differential diagnosis such as PML or MS. Accordingly, while elevated protein levels do not provide specificity needed for diagnosis, Datar and colleagues were able to show that protein levels directly correlate with both the distribution and extent of cerebral edema in PRES [7]. Thus, CSF protein levels might prove valuable for assessing the prognosis in patients suffering PRES. We employed in-depth FC to analyze the innate and adaptive immune cell compartment. Here, we observed no changes to the adaptive immune response, arguing against a central role of adaptive immunity in PRES pathophysiology. In contrast to PRES, MS or PML is characterized by clear alterations in adaptive immune responses dominated by B cells and T cells [17, 18]. In PRES, monocyte subsets displayed marked alterations and therefore point towards innate responses as a driver of PRES development.

Monocyte subsets include intermediate (CD14^++^/CD16^+^) and non-classical monocytes (CD14^+^/CD16^++^) thought to emerge sequentially from classical (CD14^+^/CD16^−^) monocytes. While subtype classification is well-established based on surface markers, the definition of distinct functional profiles to individual monocyte subtypes remains controversial: Under steady-state conditions, various studies characterized intermediate monocytes as predominant source of pro-inflammatory cytokines and potent activators of Th17 cells, [19, 20] while other studies describe non-classical monocytes as major pro-inflammatory subtype [21, 22]. Although functional redundancies prevail to be resolved, intermediate monocytes remain a potent source for the activation of pro-inflammatory pathways [23, 24]. Indeed, activation of monocytes and lymphocytes might give rise to inflammatory conditions, which can lead to endothelial dysfunction and, consequently, to interstitial damage [2]. Underlining the role of monocytes for vascular dysfunction, monocytes are implicated in the initiation and propagation of atherosclerosis [25, 26]. Recruitment and adherence of monocytes is mediated by chemokine receptors such as CXCL12 [27] and the SIRT signaling pathway [28]. While the role of innate immune cells in chronic vascular dysfunction is becoming more clear [29], the significance of these cells in acute diseases remains elusive. In the context of PRES, first characterization of pro-inflammatory intermediate monocytes as provided by this study highlights the potential role of this monocyte subset in vascular disease. However, the pathophysiological mechanism by which monocytes might mediate vascular disease remains elusive and requires further studies. Interestingly, intermediate monocytes are associated with systemic complications following cardiac surgery and are increased in advanced-stage peripheral artery occlusive disease [15], pointing towards a common mechanism of action. We hypothesize that monocyte alterations might either mediate or are evidence of “downstream” vascular dysfunction in response to a systemic challenge, such as infections, surgery, or immunosuppressant therapies. Further studies delineating the mechanism of action are required.

The importance of intermediate monocytes as a potential driver of vascular disorders in general and PRES in particular is further highlighted by the association with the length of hospital stay providing a potential use for these cells in risk stratification, as already proposed for other vascular events, such as cardiac surgery [15]. Here, similar to the present study, levels of intermediate monocytes correlated with the length of hospital stay as surrogate marker for disease severity [15]. Lastly, identification of intermediate monocytes might hold clinical value as this subpopulation differentiates PRES from PML patients as neuroinflammatory disorder presenting with similar radiographic features through readily accessible blood testing, thus, potentially improving diagnostic approaches and consequently management of PRES. In patients with diagnosis complicated by overlapping clinical and radiological findings, assessment of monocytes in PB might provide diagnostic clues guiding the initiation of appropriate therapy for PRES. As sampling occurred prior to any treatments, assessment of monocytes might be most appropriate in the initial clinical workup. However, further studies in the form of prospective trials with a larger sample size are needed for a conclusive statement.

A limitation of this study is the potential bias of patients towards severe cases due to referral bias as all patients were recruited at a major tertiary medical center. Moreover, due to the retrospective design of the study, patient selection and presence of comorbidities might be biased and a potential confounder for immunological read-out parameters. Lastly, as most PML patients included suffered from MS as underlying disease, overlapping immune mechanisms are likely shared between both cohorts and can only be partially appreciated in the present study design.

In summary, our study provides first in vivo evidence for the involvement of the innate immune response in PRES as well as the identification of an immune signature that might be of value for diagnostic approaches.

## Supplementary Information

Suppl. Fig. 1 (PNG 760 kb)

High resolution image (TIF 175 kb)

Suppl. Fig. 2 (PNG 428 kb)

High resolution image (EPS 648 kb)

Suppl. Fig. 3 (PNG 223 kb)

High resolution image (EPS 142 kb)

ESM 1 (DOCX 28 kb)

## Data Availability

Data will be shared upon reasonable request.

## Abbreviations

BCSFB: Blood-CSF-barrier
CSF: Cerebrospinal fluid
FC: Flow cytometry
MRI: Magnetic resonance imaging
MS: Multiple sclerosis
PB: Peripheral blood
PML: Progressive multifocal leukoencephalopathy
PRES: Posterior reversible encephalopathy syndrome
