# Supplementary figures and images for: The Innate Immune Response Characterizes Posterior Reversible Encephalopathy Syndrome
Source: J Clin Immunol. 2021 Apr 12;41(6):1229–40. doi: 10.1007/s10875-021-01033-3 (PMC8310851; doi:10.1007/s10875-021-01033-3)

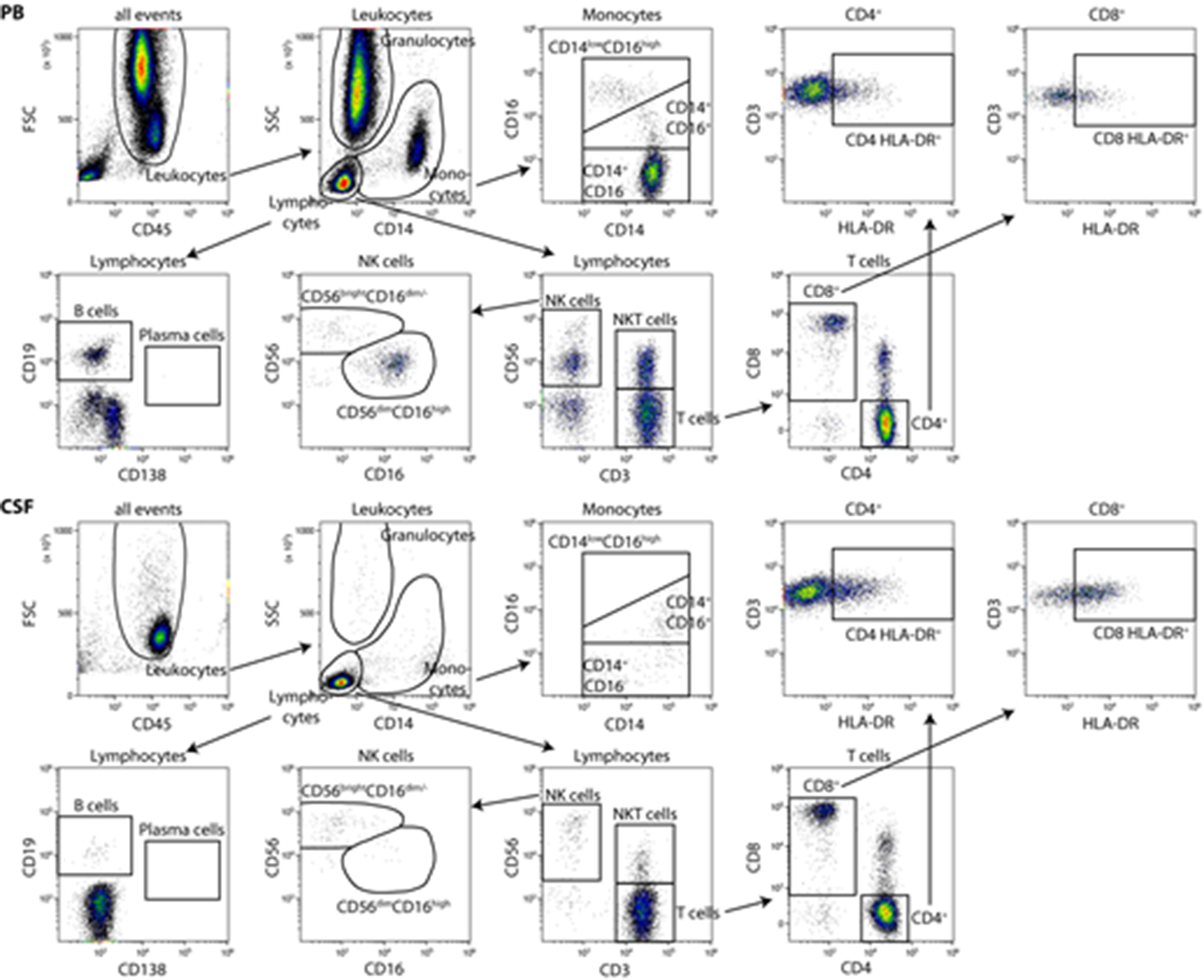

Supplement: Supplementary file 1 — (PNG 760 kb) [file 10875_2021_1033_Fig6_ESM.png]

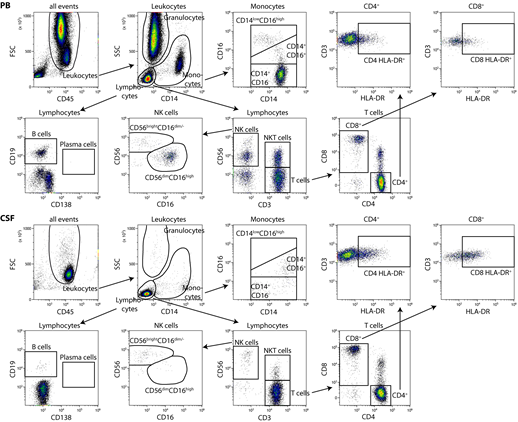

Supplement: Supplementary file 2 — High resolution image (TIF 175 kb) [file 10875_2021_1033_MOESM1_ESM.tif]

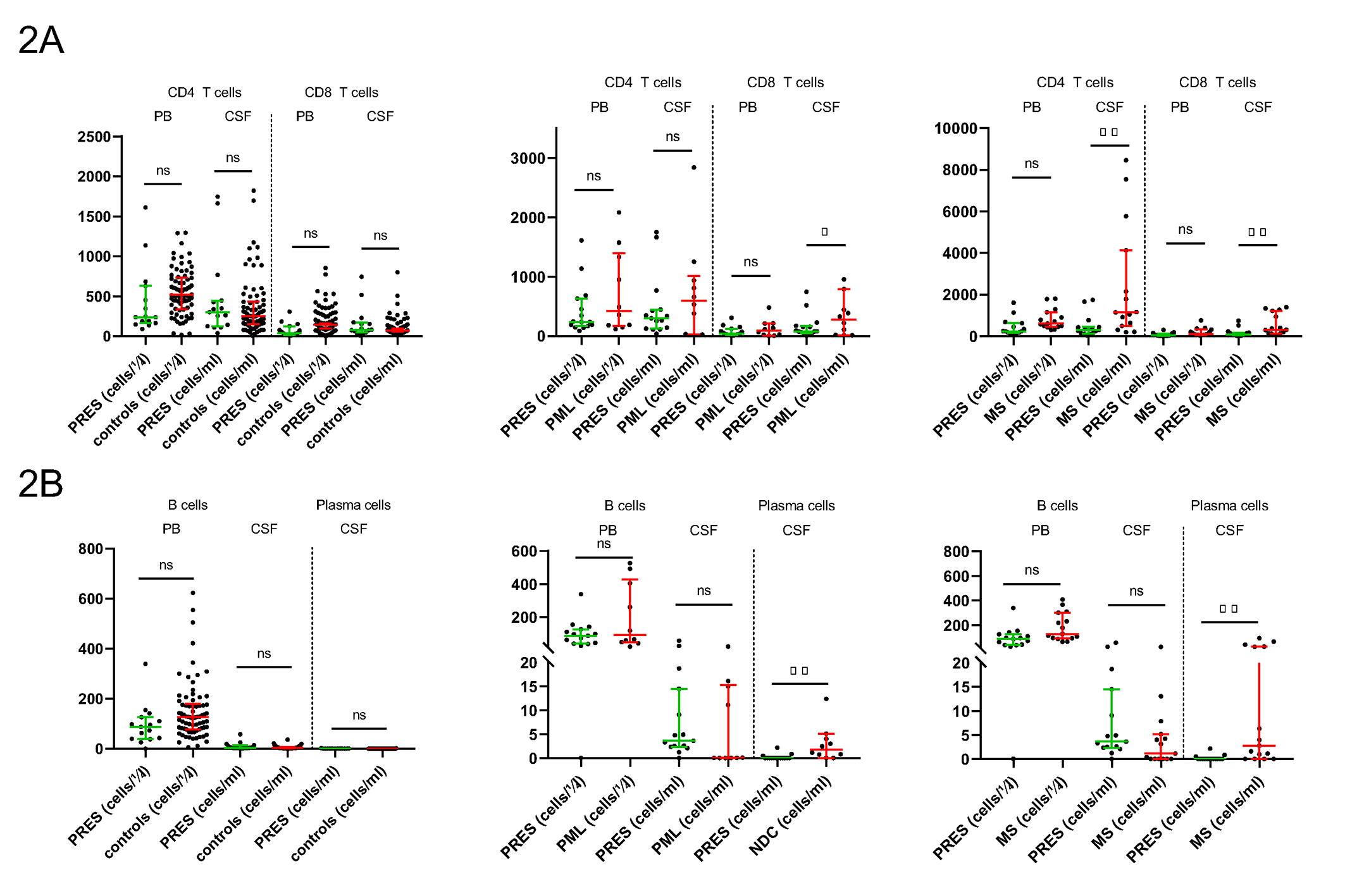

Supplement: Supplementary file 3 — (PNG 428 kb) [file 10875_2021_1033_Fig7_ESM.png]

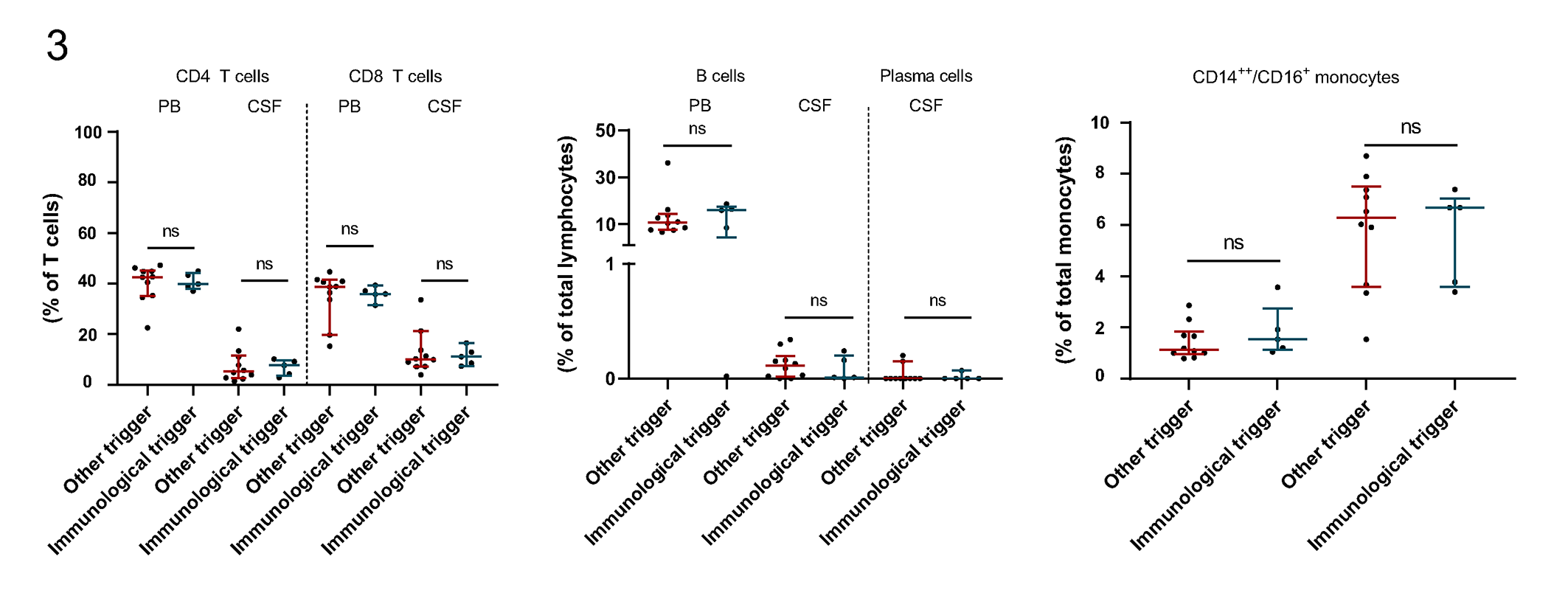

Supplement: Supplementary file 5 — (PNG 223 kb) [file 10875_2021_1033_Fig8_ESM.png]
