# Supplementary material for: The Innate Immune Response Characterizes Posterior Reversible Encephalopathy Syndrome
Source: J Clin Immunol. 2021 Apr 12;41(6):1229–40. doi: 10.1007/s10875-021-01033-3 (PMC8310851; doi:10.1007/s10875-021-01033-3)
Supplement: Supplementary file 7 — (DOCX 28 kb) [file 10875_2021_1033_MOESM4_ESM.docx]

**Suppl. Fig. 1**

**Gating strategy.** Cells from the PB and CSF (bottom) were simultaneously analyzed by flow cytometry. Total leukocytes were identified by forward scatter channel (FSC) characteristics and as CD45 expressing cells. From these, lymphocytes, monocytes, and granulocytes were selected based on side scatter channel (SSC) signal and CD14 expression. Monocytes were further differentiated into CD14^+^CD16^-^, CD14^+^CD16^+^, and CD14^low^CD16^high^ cells. Within lymphocytes, B cells were identified as CD19^+^CD138^-^ cells, whereas plasma cells were identified as CD19^low^CD138^high^ cells. Furthermore, lymphocytes were divided into CD3^+^CD56^+^ NKT cells, CD3^+^CD56^-^ T cells, and CD56^+^CD3^-^ NK cells. NK cells were further separated into CD56^dim^CD16^high^ and CD56^bright^CD16^dim/-^ NK-cell subsets. T cells were split into CD4^+^CD8^-^ and CD8^+^CD4^-^ T cells, which were further investigated for expression of HLA-DR. Percentages of leukocyte subsets refer to total leukocytes, whereas monocyte and lymphocyte subset percentages refer to total monocytes and lymphocytes, respectively.

**Suppl. Table 1**

| **Supplemental Table: Clinical characteristics of posterior reversible encephalopathy syndrome (PRES) patients** | | | | | | | |
| --- | --- | --- | --- | --- | --- | --- | --- |
| **ID** | **Age at manifestation** | **Sex** | **Predisposition diseases or trigger** | **Comorbidities** | **Therapy at manifestation**  **(immune modulatory therapy or chemotherapy)** | **Blood pressure at manifestation (systolic/diastolic)** |  |
| 1 | 80 | Female | Hypertension | Depression, osteoporosis | None | 220/120 |  |
| 2 | 72 | Male | Chronic renal insufficiency | Anaemia | None | 180/100 |  |
| 3 | 52 | Female | None | Smoking, chronic pulmonary obstruction disease | None | 120/80 |  |
| 4 | 76 | Female | Hypertension, chronic renal insufficiency | Chronic bronchitis, diabetes mellitus, heart insufficiency | None | 210/110 |  |
| 5 | 56 | Female | Hypertension | Atrial fibrillation, herpes zoster | None | 200/110 |  |
| 5 | 72 | Female | Hypertension, ovarian cancer | Morbus Parkinson | Carboplatin, paclitaxel | 150/90 |  |
| 6 | 52 | Female | None | Smoking | None | 130/80 |  |
| 7 | 76 | Male | Pneumonia | Congestive heart failure, diabetes mellitus | None | 140/90 |  |
| 8 | 81 | Female | Sepsis, pneumonia | None | None | 130/65 |  |
| 9 | 52 | Male | Hypertension, pneumonia | Asthma, aortic valve insufficiency | None | 160/60 |  |
| 10 | 43 | Male | None | Smoking, chronic pulmonary obstruction disease | None | 130/95 |  |
| 11 | 58 | Female | Chronic renal insufficiency | Diabetes mellitus, depression, humerus fracture | None | 140/90 |  |
| 12 | 64 | Male | Hypertension | None | None | 180/105 |  |
| 13 | 78 | Male | Sepsis | Diabetes mellitus | None | 110/70 |  |
| 14 | 31 | Female | Chronic renal insufficiency | Unilateral renal agenesis, anaemia | None | 120/70 |  |
| 15 | 52 | Male | None | Diabetes mellitus | None | 130/80 |  |

**Suppl. Table 1.** Clinical and demographic data of PRES patients included in the analysis.

**Suppl. Table 2**

| **Supplemental Table: Clinical characteristics of multiple sclerosis (MS) and progressive multifocal leukoencephalopathy (PML) patients** | | | | | |
| --- | --- | --- | --- | --- | --- |
| **ID** | **Age at manifestation** | **Sex** | **Disease** | **Comorbidities** | **Therapy at manifestation**  **(immune therapy)** |
| 1 | 49 | Male | MS | None | None |
| 2 | 42 | Male | MS | None | None |
| 3 | 48 | Female | MS | None | None |
| 4 | 49 | Male | MS | Hypertension, depression | None |
| 5 | 40 | Female | MS | Hypothyroidism, asthma | None |
| 5 | 42 | Female | MS | None | None |
| 6 | 33 | Female | MS | None | None |
| 7 | 52 | Male | MS | None | None |
| 8 | 32 | Female | MS | Asthma | None |
| 9 | 32 | Female | MS | Smoking, | None |
| 10 | 32 | Female | MS | None | None |
| 11 | 48 | Male | MS | None | None |
| 12 | 47 | Male | MS | Depression | None |
| 13 | 46 | Male | MS | Coeliac disease | None |
| 14 | 46 | Female | MS | None | None |
| 15 | 42 | Female | MS | Smoking | None |
| 16 | 51 | Male | MS (PML) | Hypertension | Natalizumab |
| 17 | 44 | Male | MS (PML) | None | Natalizumab |
| 18 | 41 | Female | MS (PML) | None | Natalizumab |
| 19 | 42 | Female | MS (PML) | Hypothyroidism, chronic venous insufficiency | Natalizumab |
| 20 | 50 | Male | MS (PML) | Gout, hypertension | Fingolimod |
| 21 | 41 | Female | MS (PML) | None | Natalizumab |
| 22 | 20 | Female | MS (PML) | None | Natalizumab |
| 23 | 74 | Male | Psoriasis (PML) | Hypertension, dyslipidaemia, smoking | Dimethyl fumarate |
| 24 | 69 | Male | Rheumatoid arthritis (PML) | Hypertension, atrial fibrillation, congestive heart failure, smoking, depression | Dimethyl fumarate |
| 25 | 22 | Female | MS (PML) | Hypothyroidism | Natalizumab |

**Suppl. Table 2.** Clinical and demographic data of MS and PML patients included in the analysis.

**Suppl. Fig. 2**

**Absolute numbers of adaptive immune cells.** (A) Absolute cell number for CD4^+^ and CD8^+^ T cells determined by Mann-Whitney test for control cohort (left), PML (middle) and MS (right). (B) Absolute cell number for B cells and plasma cells determined by Mann-Whitney test for control cohort (left, PML (middle) and MS (right). For each comparison, PB is left, CSF is right. Error bars display median (IQR). Units were scaled to allow for better presentation. Abbreviations: CSF = cerebrospinal fluid, MS = multiple sclerosis, PB = peripheral blood, PML = progressive multifocal leukoencephalopathy, PRES = posterior reversible encephalopathy syndrome. *** p < 0.001, ** p < 0.01, * p < 0.05

**Suppl. Fig. 3**

**Influence of immunological triggers** Relative cell number for CD4^+^ and CD8^+^ T cells, B cells and plasma cells as well as intermediate monocytes determined by Mann-Whitney test for immunological trigger (PRES likely triggered by infections or immune suppression) and other triggers (PRES likely triggered by other causes). For each comparison, PB is left, CSF is right. Error bars display median (IQR). Abbreviations: CSF = cerebrospinal fluid, PB = peripheral blood, PRES = posterior reversible encephalopathy syndrome. We observed no significant differences.
